# Impact of age and mismatch repair status on survival in colorectal cancer

**DOI:** 10.1002/cam4.1007

**Published:** 2017-03-27

**Authors:** Pan Li, Zhi‐Tao Xiao, Todd A. Braciak, Qing‐Jian Ou, Gong Chen, Fuat S. Oduncu

**Affiliations:** ^1^ Department of hematology and oncologyMedizinische Klinik und Poliklinik IVLudwig Maximilians UniversityMunichGermany; ^2^ Department of Colorectal SurgeryState Key Laboratory of Oncology in South ChinaSun Yat‐sen University Cancer CenterGuangzhouChina

**Keywords:** Age, biomarker, colorectal cancer, MMR status, prognosis

## Abstract

Previous studies have suggested that deficiencies in mismatch repair genes (dMMR) often occur in patients with colorectal cancer (CRC) and contribute to disease etiology. Here, we looked for a correlation of MMR status to disease outcomes from a large number of Chinese CRC patients stratified by the age of onset of disease. A total of 2233 CRC patients were analyzed and tissue biopsies of surgically removed tumors scored for MMR gene status. The patient distribution after classification consisted of 188 younger aged patients (20–39 years of age), 1024 middle aged patients (40–59 years of age), and 1020 older aged patients (60–85 years of age). In this analysis, the expression of four MMR genes was assessed by immunohistochemistry (IHC). We found that the young group of CRC patients with dMMR had higher overall survival (OS) than the young group of patients with proficient MMR (pMMR) (77% vs. 56%, *P *=* *0.03). Middle‐aged patients with dMMR also had higher OS than middle‐aged group patients with pMMR (78% vs. 68%, *P *=* *0.012). However, we found no statistical difference in OS between dMMR and pMMR status in the older group of patients (75% vs. 71%, *P *=* *0.224). Finally, the middle‐ and older‐aged group set of patients had higher OS than the young group of patients (69% vs. 71% vs. 59%, *P *=* *0.008). These data demonstrated that the age of disease onset can be an important factor to help evaluate the prognosis of CRC when combined with the analysis of MMR status within tumor biopsied tissue.

## Introduction

The development of colorectal cancer (CRC) can be a slow process, with early stages of disease often presenting with no clinical symptoms to the patient. It has been indicated that most CRCs are adenocarcinomas arising from noncancerous adenomatous polyps 1, 2. Like many other types of cancer, CRC has been found to have an associated higher incidence with increasing age. The risk of CRC has a marked increase in occurrence after reaching 40 years of age and incidence continues to increase even more rapidly after this age. Overall, the risk of CRC doubles with each succeeding decade of age, and continues to rise exponentially in incidence with age 3.

Little is known about the precise biochemical mechanisms responsible for the rise in CRC with aging. Many possible causes for this increase in CRC incidence have been suggested. One model of CRC progression proposed by Vogelstein et al. indicates that malignancy arises as a result of accumulation of mutations in tumor suppressor genes and oncogenes 4. Indeed, the molecular profiles of CRC patients have been shown to differ between various age groups of patients examined 5, 6, 7. Whether patients within these various age groups have a different biological behavior including MMR activity remains controversial.

MMR genes correct mismatched nucleotides and insertion–deletion loops (IDLs) in DNA caused by polymerase errors, chemical modifications, and recombination between heterologous DNA sequences 8. It was previously demonstrated that stage II CRC patients with dMMR have a better prognosis and it was indicated that some patients may actually be harmed by 5‐FU treatment 9. More recent studies have found that CRC tumors with dMMR were more prevalent in younger patients 10, 11. Despite these early findings about MMR status, there still has been no conclusive study to prove an association with overall survival (OS) for age of disease onset and MMR status in CRC patients.

In this study, we sought to find if difference in MMR status based on age could be useful to identify groups of patients that would have better disease outcomes. Following the recommendations of the EGAPP 12, we performed systematic immunohistochemistry (IHC) screening of CRC tumor tissues looking for microsatellite instability of patients. Here, we had access to a large number of Chinese CRC patients who were operated on to remove their primary tumors and whose tissues were assessed in our institute since 2011. Our findings indicate that the combined analysis of age of onset and MMR status could provide some prognostic information about these CRC patient outcomes.

## Materials and Methods

### Patients

The ethics committee of Sun Yat‐sen University Cancer Center approved this study and informed consents for all patients were obtained at the beginning of the study. A total of 4500 patients with histologically confirmed CRC tumors were recruited from the Sun Yat‐sen University cancer center between May 2011 and May 2016 for this study. Clinical and familial histories for each of these patients were reviewed. From these recruited patients, 2233 cases were finally selected for analysis after applying strict exclusion criteria that included: age less than 18 years and older than 85 years, severe complication, multiprimary cancer, synchronous and metachronous CRC, family history (first‐degree and second‐degree relatives had any kind of cancer), familial adenomatous polyposis, death not due to tumor‐related reason, and incomplete follow‐up record were not included for study. The primary tumor location site was categorized as right colon if the tumor was located above the splenic flexure, and left colon if it was located at or below the splenic flexure or as from the rectum. The median follow‐up on surviving patients was 4.3 years.

### Treatment and follow‐up

Stage I (T1–2 N0) and stage II (T3–4 N0) CRC patients without high‐risk clinical features (e.g., T4 stage, bowel perforation or clinical bowel obstruction, inadequate lymph node sampling, poorly differentiated histology) were treated with radical surgery or endoscopic removal of the tumor alone. Stage II (T3–4 N0) CRC patients with high‐risk clinical features were recommended to follow the capecitabine+oxaliplatin (XELODA) treatment regimen. Stage III (Tx N1–2) patients were designated to receive radical surgery and 12 cycles of adjuvant mFOLFOX/XELOX regimen treatment within a 6‐month period. All stage IV (Tx Nx M1) patients received palliative surgery or radical surgery. The first‐line treatment for stage IV CRC was mFOLFOX/FOLFIRI (folinic acid+5‐FU+irinotecan) chemotherapy regimen. Patient clinical responses were evaluated in accordance with the RECIST guidelines. After surgery, tumor recurrence was determined by physical examination, serum carcinoembryonic antigen (CEA) assay, or abdominal and thoracic imaging taken every 3–6 months for the following 3 years after initial therapy, then every 6 months for the next 2 years, and finally followed by annual checkup. In addition, Sun Yat‐sen University cancer center has an independent follow‐up department. The colleagues call the patients or the family members regularly and register the survival status and health condition. The cutoff date of analysis for inclusion in this study was May 2016.

### Immunohistochemistry

**Figure 1:**
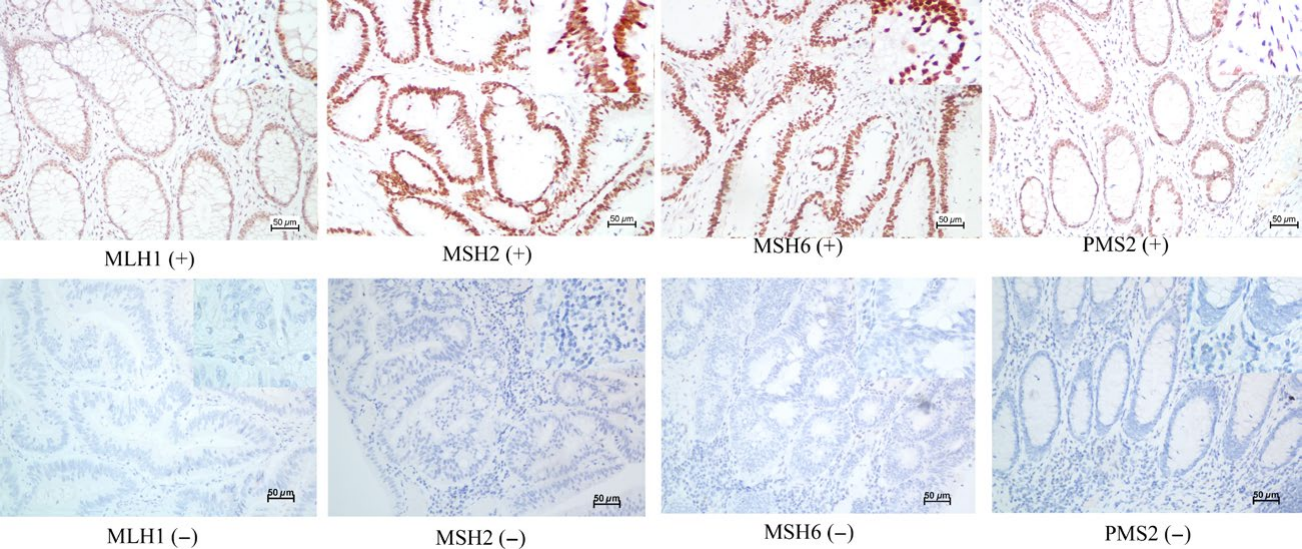
Illustrative immunostainings. Positive (upper panel) and negative (lower panel) for MLH1, MSH2, MSH6, and PMS2.

Blocks of formalin‐fixed, paraffin‐embedded CRC adenocarcinoma tissue comprising an area of normal colorectal mucosa adjacent to the tumor were selected in each case. Cases with complete nuclear loss of MMR expression in invasive tumor cells but with retained expression in inflammatory cells and/or adjacent normal tissue as positive controls were considered MMR deficient. Staining was performed using the following primary antibodies: mouse anti‐human mutL homolog 1 (MLH1) (dilution 1:150, clone OTI1C1, zhongshan jiqiao, Beijing), rabbit anti‐human mutS homolog2 (MSH2) (dilution 1:100, clone ZA0622, zhongshan jiqiao, Beijing, mouse anti‐human mutS homolog 6 (MSH6) (dilution 1:150, clone OTI5D1, zhongshan jiqiao, Beijing), and mouse anti‐human postmeiotic segregation increased 2 (PMS2) (dilution 1:150, clone OTI2G5, zhongshan jiqiao, Beijing). Whole‐tissue sections were analyzed independently by two pathologists and were blinded to any of the patients’ clinical characteristics. Any discordant cases were reviewed by a supplementary pathologist in order to reach a consensus on the tumor characterization. Illustrative immunostainings of recovered CRC tumor sections are shown in Figure 1.

### Statistical analysis

Patient data are described in frequencies (percentages) of cases with the given phenotype. Differences in distributions between the variables examined were assessed by the χ^2^ or the Fisher's exact test. The primary end point of the study was OS, defined as the time elapsed from the date of surgery until the tumor‐induced death of the patient. Surviving patients were censored on the last follow‐up date. Median follow‐up and the 95% CI were calculated using the reverse Kaplan–Meier method. The survival curve was estimated with the Kaplan–Meier method and compared using the log‐rank test. The score and likelihood ratio test *P‐*values were used to test the statistical significance of each covariate in the univariate and multivariable Cox models, respectively. All statistical tests were two‐sided, and *P‐*values less than or equal to 0.05 were considered statistically significant. Statistical analyses were performed using SPSS software.

## Results

A total of 2001 (89.6%) of all CRC specimens examined showed retained expression in tumor cells for MLH1, MSH2, MSH6, and PMS2 proteins. In comparison, loss of expression in at least one of the four MMR genes was found to occur in 232 of 2233 patients analyzed representing a dMMR status for 10.4% of the CRC patients examined. The patients analyzed included 188 patients (8.4%) classified as young (20–39 years old), 1024 (45.9%) as middle‐aged patients (40–59 years old), and 1020 (45.7%) as older‐aged patients (60–85 years old). Their median age at diagnosis was 58 years (ranged 18–85 years). The patient demographics and tumor characteristics by age are listed in Table 1.

**Table 1:** Clinicopathological characteristics of the patients

Characteristics	Ages (*n*/%)			*P‐*value
	20–39 years (188/8.4)	40–59 years (1024/45.9)	60–85 years (1021/45.7)	
Gender				<0.001
Male	84 (3.8)	599 (26.8)	633 (28.3)	
Female	104 (4.7)	425 (19.0)	388 (17.4)	
Location				0.228
Right colon	52 (2.3)	243 (10.9)	226 (10.1)	
Left colon	59 (2.6)	323 (14.5)	358 (16.0)	
Rectum	77 (3.4)	458 (20.5)	437 (19.6)	
Pathology				0.095
G1	18 (0.8)	48 (2.1)	38 (1.7)	
G2	152 (6.8)	919 (41.2)	931 (41.7)	
G3	0 (0.0)	5 (0.2)	13 (0.6)	
Mucinous	13 (0.6)	46 (2.1)	36 (1.6)	
Signet‐ring	5 (0.2)	6 (0.3)	3 (0.1)	
Stage				<0.001
I	13 (0.6)	129 (5.8)	189 (8.5)	
IIA	57 (2.6)	273 (12.2)	280 (12.5)	
IIB	13 (0.6)	119 (5.3)	93 (4.2)	
IIC	3 (0.1)	17 (0.8)	22 (1.0)	
IIIA	6 (0.3)	25 (1.1)	24 (1.1)	
IIIB	30 (1.3)	209 (9.4)	213 (9.5)	
IIIC	14 (0.6)	47 (2.1)	31 (1.4)	
IVA	28 (1.3)	123 (5.5)	114 (5.1)	
IVB	24 (1.1)	82 (3.7)	55 (2.5)	
MMR status				<0.001
dMMR	26 (1.2)	125 (5.6)	81 (3.6)	
pMMR	162 (7.3)	899 (40.3)	940 (42.1)	
Alive				0.001
Yes	111 (5.0)	707 (31.7)	726 (32.5)	
No	77 (3.4)	317 (14.2)	295 (13.2)	

**Table 2:** Univariate analysis of overall survival

		95% CI		
Variable	HR	Lower	Upper	*P*‐value
Tumor stage	0.19	0.16	0.23	<0.001
Gender	0.96	0.82	1.11	0.561
Tumor location	0.92	0.83	1.01	0.064
Pathology	0.97	0.84	1.12	0.700
MMR status	1.56	1.18	2.06	0.002

In addition, we found that 55.3% of the younger patient group were female. With regard to dMMR status, we found that tumors with dMMR status tended to be noticed in higher incidence in younger patients (13.8%) compared to middle‐age (12.2%) and older patients (7.9%) (*P < *0.001). In univariate analysis, the MMR status (HR: 1.56, 95% CI: 1.18–2.06, *P *=* *0.002) and tumor stage (HR: 0.19, 95% CI: 0.16–0.23, *P *<* *0.001) showed statistical significance. However, among the variables analyzed in the multivariate Cox model, only tumor stage (HR: 0.20, 95% CI: 0.17–0.24, *P *<* *0.001) was shown to be significantly associated with OS regarding the age of disease onset. We found that gender (HR: 0.96, 95% CI: 0.82–1.11, *P *=* *0.561), tumor location (HR: 0.92, 95% CI: 0.83–1.01, *P *=* *0.064), and pathological differentiation (HR: 0.97, 95% CI: 0.84–1.12, *P *=* *0.700) showed no statistical differences by univariate Cox analysis (*P *>* *0.05) regarding the age of disease onset.

**Figure 2:**
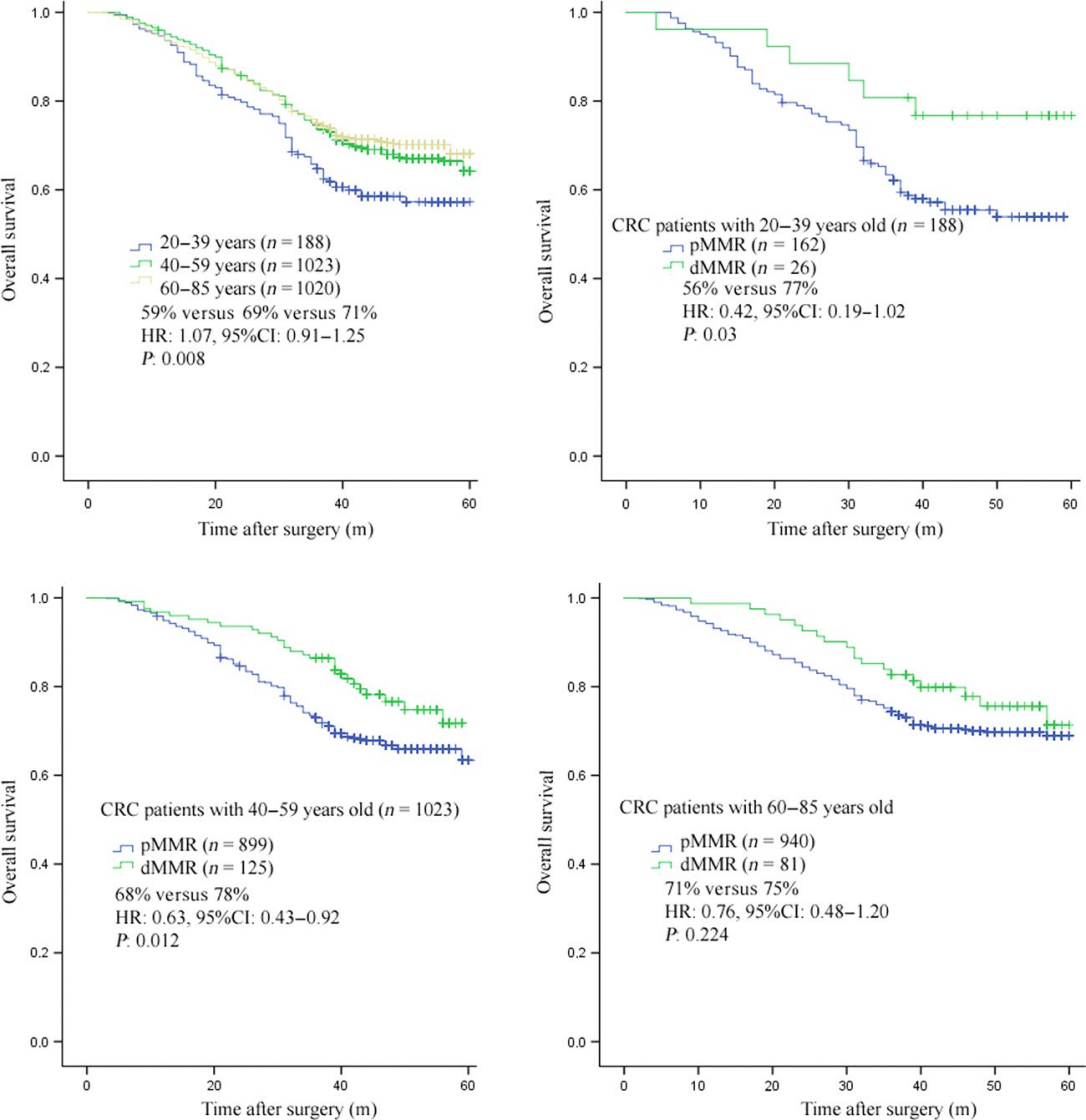
Overall survival (OS) according to MMR status and age in colorectal carcinoma (CRC) stage I‐IV.

Our most important finding in this study was that we could find an association for dMMR status and age of disease onset to OS for our Chinese CRC patients examined. As part of this analysis, we found that age alone had associations with OS. Here, middle‐ and older‐aged patients had higher OS than younger‐aged grouped patients (69% vs. 71% vs. 59%, HR: 1.07, 95% confidence interval (CI): 0.91–1.25, *P *=* *0.008). In our results, the middle‐ (69%) and older‐aged (71%) patients have similar OS with no significant difference. However, when the middle‐ and older‐aged patients are compared with younger‐aged patients (59%), statistical significance is shown. When age was analyzed in conjunction with MMR status, we found that younger CRC patients with dMMR had higher OS than young patients with proficient MMR (pMMR) (77% vs. 56%, HR: 0.42, 95% CI: 0.19–1.02, *P *=* *0.03). Likewise, we found that middle‐aged patients with dMMR also had higher OS than middle‐aged patients with pMMR (78% vs. 68%, HR: 0.63, 95% CI: 0.43–0.92, *P *=* *0.012). This association of age with dMMR did not hold for older‐age group patients. Here, we found no statistical difference in OS between dMMR and pMMR status for older‐aged CRC patients (75% vs. 71%, HR: 0.76, 95% CI: 0.48–1.20, *P *=* *0.224). Patient survival plots with regard to tumor location and MMR status are shown in Figure 2.

## Discussion

Multiple risk factors contribute to the capacity of an individual to develop CRC. Disturbingly, younger‐aged patients are more likely to be diagnosed with later stage disease when their cancers are discovered 13. However, it is unclear whether this reflects differing biology between young versus older patients or simply that a low rate of CRC screening is performed at this young age. In our present Chinese population‐based CRC study, we looked to see if age and MMR status had an impact on the survival of these CRC patients.

It was previously reported that younger patients have a higher prevalence of mucinous or signet‐ring types of carcinoma 14, 15. Yet here, in our study, we found that pathological differentiation showed no statistical difference to OS (*P *>* *0.05). Another study demonstrating a linkage to age and CRC demonstrated that the age at diagnosis of inflammatory bowel disease was an indicator of early development of CRC in inflammatory bowel disease patients 7.

In younger patients, CRC tend to present more commonly as stage III or IV disease, which may reflect differing biology in younger‐aged CRC patients, but could also be reflective of later diagnosis because of the rarity of this condition in that age group, and/or less surveillance in general of cancers in this age group 13. In concordance with this previous study, here, we also found that younger patients were more likely to present as stage IIIB or IVB disease.

The evidence is increasing to indicate that the molecular profiles of CRC cells can differ in various age groups of patients and that this will influence the given patient's disease outcome and response to therapy 5, 6, 7. It was reported that CRC patients above the age of 50 showed decreased Bax/Bcl‐2 ratios that might differentially control tumor cell apoptosis between the various age groups of patients 5. Also, it has been shown that peripheral blood leukocyte (PBL) telomere length varies according to the age of CRC onset, perhaps impacting immune cell functions differentially 6. With regard to MMR status, a more recent set of studies found that dMMR was more prevalent in younger CRC patients 10, 11. Likewise, we found that tumors with a dMMR status tended to be more prevalent in the younger group of patients (13.8%) versus the middle‐ (12.2%) or older‐aged patients (7.9%) (*P < *0.001).

MMR corrects mismatched nucleotides and insertion–deletion loops (IDLs) in DNA caused by polymerase errors, chemical modifications, and recombination between heterologous DNA sequences 8. It was demonstrated that stage II patients with dMMR have a better prognosis and may actually be harmed by 5‐FU treatment 9. In our study, young CRC patients with dMMR had higher OS than young patients with pMMR (*P *=* *0.03). We also found that middle‐aged patients with dMMR also had higher OS than middle‐aged patients with pMMR (*P *=* *0.012). However, we found no statistical difference in OS between dMMR and pMMR status in older‐aged patients (*P *=* *0.224). While not yet known, these differences in disease response may be based upon the molecular profile or epigenetics among these various aged patients.

Overall, the notion that age can be a significant prognostic factor in CRC has been somewhat controversial. Various studies have reported poorer prognosis for younger patients with CRC 16, 17, 18, 19. Yet, other authors have demonstrated that younger patients with CRC surgically treated appeared to have a higher cancer‐specific survival rate than elderly ones 13, 19, 20. In our study here, we found that middle‐ and older‐aged patients had higher OS than young patients (*P *=* *0.008), indicating that again there is some link to age and CRC patient disease response.

In conclusion, the observation that cancer‐related mortality did not decrease with increasing age exemplified that, although elderly patients have a shorter life expectancy based on their age and preexistent conditions, they did still benefit from cancer treatment. The combination of age and MMR was a significant predictor of overall survival in our study, reflecting the importance of optimizing patient beyond treatment. The potential efficacy of age‐tailored and molecular profiled interventions as components of personalized care clearly need to be investigated further in future studies.

## Conflict of Interest

The authors declare that they have no competing interests.
